# Consumer perceptions of the sale of tobacco products in pharmacies and grocery stores among U.S. adults

**DOI:** 10.1186/1756-0500-6-261

**Published:** 2013-07-09

**Authors:** Pallavi Patwardhan, Robert McMillen, Jonathan P Winickoff

**Affiliations:** 1 Schroeder Institute for Tobacco Research and Policy Studies, Legacy, 1724 Massachusetts Ave, NW, Washington, DC 20036, USA; 2 Department of Psychology and Social Science Research Center, Mississippi State University, One ResearchPark, Suite 103, Starkville, MS 39762, USA; 3 Julius B. Richmond Center of Excellence, American Academy of Pediatrics, 141 Northwest Point Boulevard, Elk Grove Village, IL 60007, USA; 4 Center for Child and Adolescent Health Research and Policy, Massachusetts General Hospital, 100 Cambridge Street, Suite 1542a, Boston, MA 02114, USA

**Keywords:** Pharmacies, Consumer perceptions, Tobacco sales, Survey research

## Abstract

**Background:**

Pharmacy-based tobacco sales are a rapidly increasing segment of the U.S. retail tobacco market. Growing evidence links easy access to tobacco retail outlets such as pharmacies to increased tobacco use. This mixed-mode survey was the first to employ a nationally representative sample of consumers (n = 3057) to explore their opinions on sale of tobacco products in pharmacies and grocery stores.

**Results:**

The majority reported that sale of tobacco products should be either ‘allowed if products hidden from view’ (29.9%, 25.6%) or ‘not allowed at all’ (24.0%, 31.3%) in grocery stores and pharmacies, respectively. Significantly fewer smokers, compared to non-smokers, reported agreement on point-of-sale restrictions on sales of tobacco products (grocery stores: 27.1% vs. 59.6%, p < .01; pharmacy: 32.8% vs. 62.0%, p < .01). Opinions also varied significantly by demographic characteristics and factors such as presence of a child in the household and urban/rural location of residence.

**Conclusions:**

Overall, a majority of consumers surveyed either supported banning sales of tobacco in grocery stores and pharmacies or allowing sales only if the products are hidden from direct view. Both policy changes would represent a departure from the status quo. Consistent with the views of practicing pharmacists and professional pharmacy organizations, consumers are also largely supportive of more restrictive policies.

## Background

Tobacco use remains the number one preventable cause of morbidity and mortality in the United States [1]. Responsible for 30% of all cancers [2], as well as respiratory, cardiovascular, and other chronic diseases, tobacco use results in billions of dollars in economic loss to society. During 2000–2004, the total economic burden of smoking in the US alone was estimated to be $193 billion annually from direct healthcare expenditures ($96 billion) and productivity losses ($97 billion) [3].Despite several decades of falling smoking prevalence rates, the CDC now warns of a stalled decline in smoking prevalence [4].

Among the various factors that influence tobacco use patterns and behaviors, there is evidence that suggests easy access to retail tobacco outlets might support higher tobacco consumption among youth [5-8]. The 2007 Institute of Medicine reports that limiting youth access to tobacco products is an essential component of tobacco control activities [9]. Tobacco companies, on the other hand, have spent 92% of their total marketing expenditures ($110 billion) since 1998, to promote and advertise their products in retail environments including pharmacies and grocery stores. From 1998 to 2008, annual tobacco spending in the retail environment increased from $5.4 billion to $9.8 billion, indicating the importance of point-of-sale marketing to the tobacco industry [10]. This advertising strategy exposes youth to tobacco marketing in grocery stores and contradicts the health orientation of pharmacies.

Although the number of independently owned pharmacies selling tobacco products decreased, chain pharmacies continue to sell cigarettes and other related products. Tobacco companies encourage product placement in prime retail locations [11], while pharmacy-based tobacco sales have in fact increased from 2005 to 2009 [12]. In contrast, practicing pharmacists and professional pharmacy organizations across the globe have consistently and strongly opposed the sale of tobacco products in such settings [13-20].

There is a growing interest among public health advocates and regulators to implement local and state level ordinances and to pass bills that would completely ban tobacco sales from pharmacies and grocery chains that have pharmacies in them [21-24]. The city of San Francisco is among the early few that have implemented a ban on the sale of tobacco products in community-based pharmacies including grocery stores [24]. This regulation drew praise from supporters of public health and the pharmacy profession. At the same time it also led to companies such as Safeway, Walgreens, and Philip Morris filing suit against the City citing inconsistent pharmacy-related business restrictions [25-27].

A study of perceptions and opinions of consumers potential restrictions can inform advocacy and policy decisions. A limited number of studies have explored consumer perceptions regarding the sale of tobacco in pharmacies and grocery stores, and the samples employed have been small and restricted to certain geographic regions thus limiting generalizability [15,28-30]. National consumer perceptions in this area remain unknown. As businesses, pharmacies closely attend to their customer preferences. This paper is the first to our knowledge that presents data from a nationally representative sample of U.S. consumers regarding their views on tobacco sales in pharmacies and grocery stores.

## Methods

Dual-frame surveys representing national probability samples of adults were administered in 2011. The design included an Random Digit Dialing (RDD) frame and an internet panel frame developed from a probability sample of U.S. adults, in order to reduce non-coverage issues arising from wireless substitution. Wireless substitution of cell phones for landlines continues to increase, and 35.8% of U.S. households are currently wireless only [31]. The RDD frame included households with listed and unlisted landline telephones; five attempts were made to contact those selected adults who were not home. The Survey Research Laboratory at Mississippi State University’s Social Science Research Center administered the surveys via computer-assisted telephone interviews to respondents in this frame. The probability-based panel frame included an online survey conducted by Knowledge Networks, administered to a randomly selected sample from a nationally representative research panel [32,33]. This panel is based on a sampling frame which includes both listed and unlisted numbers, those without a landline telephone, and does not accept self-selected volunteers [32,33], and provides sample coverage for 99% of U.S. households [34]. Surveys were administered to both frames from October to November 2011. The Institutional Review Board at Mississippi State University approved this study on July 30, 2011. Informed verbal consent was obtained and the IRB provided a waiver of documentation of the written consent process. More detailed methods have been previously published [35].

Data were weighted to adjust for age, race, gender, and region, as well as frame overlap among internet panel respondents who also had a landline telephone and were therefore also eligible for the RDD frame.

### Consumer opinions

Respondents were asked “In grocery stores, should the sale of tobacco products be allowed; allowed, but only if the products are hidden from view; or not allowed at all?” The question was repeated for pharmacies and drug stores^a^. Respondents were then asked, “I would prefer to get my medications from a pharmacy that doesn’t also sell tobacco products. Do you strongly agree, agree, disagree, or strongly disagree?” Respondents who disagreed with this item or reported “don’t know” were asked, “If my doctor recommended a pharmacy because they didn‘t sell tobacco products, I would get my medication there. Do you strongly agree, agree, disagree, or strongly disagree?” These items were dichotomized for the analyses in this study.

### Self-reported smoking

Respondents were asked, “Have you smoked at least 100 cigarettes in your entire life?” Respondents who reported that they had were then asked, “Do you now smoke cigarettes every day, some days, or not at all?” Respondents who reported that they have smoked at least 100 cigarettes and now smoke every day or some days were categorized as current smokers.

### Analyses

Descriptive and bivariate analyses examined overall and subpopulation consumer opinions. We used bivariate chi-square analyses and multivariable logistic regression to explore demographic factors associated with tighter restrictions on selling tobacco products in pharmacies and grocery stores. In order to explore the possibility that respondents from the internet panel frame might develop a response bias as they gained more experience with responding to surveys, we performed a separate set of logistic regression analyses to examine the relationship of length of time on panel with consumer opinions in the panel frame.

## Results

In the RDD frame, of 2,282 eligible respondents contacted, 1,500 (63%) completed surveys [35]. For the probability-based panel frame, 2,476 panelists were randomly drawn from the probability panel; 1,597 responded to the invitation, yielding a final stage completion rate of 65% percent [36,37]. Length of time on the panel for the probability-based panel frame ranged from 0.18 to 11.9 years, with a median length of time on the panel of 1.4 years. Table 1 shows the demographic characteristics of the overall sample.

**Table 1:** Survey sample characteristics, (N = 3,059)

**Sample characteristic**	**n(%)**
Smoking Status	
Current smoker	546 (18.1)
Nonsmoker	2,472 (81.9)
Gender	
Female	1,582 (51.7)
Male	1,476 (48.3)
Race	
White	2,124 (69.5)
African American	347 (11.3)
Other	588 (19.2)
Age	
18-29 years	533 (18.3)
30-44 years	896 (30.7)
45-59 years	884 (30.3)
60+ years	599 (20.8)
Region	
Northeast	395 (12.9)
Midwest	579 (18.9)
South	1,137 (37.2)
West	948 (31.0)
Location of residence	
Urban	2,316 (77.1)
Rural	690 (22.9)
Children in household	
Yes	1,138 (37.65)
No	1,920 (62.8)

The majority reported that sale of tobacco products should be restricted in grocery stores and pharmacies. For grocery stores, 29.9% of consumers believed that tobacco products should be ‘allowed if products hidden from view’ and 24.0% believed that these products should ‘not allowed at all’. Similar levels of support were detected for pharmacies; 25.6% reported ‘allowed if products hidden from view’ and 31.3% reported ‘not allowed at all’^b^. Significantly fewer smokers, compared to non-smokers, reported agreement on point-of-sale restrictions on tobacco products (grocery stores: 27.1% vs. 59.6%, p < .01; pharmacy: 32.8% vs. 62.0%, p < 0.01). Bivariate chi-square analyses also revealed statistically significant differences in support for point-of-sale restrictions by parental status, age, region, sex, and race (see Tables 2 and 3). These characteristics remained statistically significant in multivariable analyses (see Table 4).

**Table 2:** Consumer views regarding point-of-sale restrictions on tobacco products in grocery stores

	**Allowed**	**Allowed, but only if the products are hidden from view**	**Not allowed at all**	**p**
**Overall**, n = 2,993	46.1%	29.9%	24.0%	
**Smoking status**				p < .001
Current smokers	72.9%	19.1%	8.0%	
Nonsmokers	40.3%	32.2%	27.4%	
**Children**				ns
Child in home	43.8%	31.9%	24.3%	
No children in home	47.5%	28.6%	23.9%	
**Age**				p < .001
Age 18-29	46.6%	32.0%	21.3%	
Age 30-44	48.7%	26.1%	25.1%	
Age 45-59	48.5%	32.6%	18.8%	
Age 60+	39.7%	29.9%	30.4%	
**Region**				p < .001
Northeast	37.6%	33.5%	28.9%	
Midwest	50.8%	28.0%	21.2%	
South	47.7%	26.8%	25.5%	
West	45.1%	33.1%	21.8%	
**Rural/Urban**				ns
Rural	49.3%	28.4%	22.3%	
Urban	45.7%	30.1%	24.2%	
**Sex**	*ns*			p < .001
Male	53.0%	26.3%	20.7%	
Female	39.8%	33.2%	27.1%	
**Race**	p < .01			p < .001
White	50.0%	28.5%	21.5%	
African-American	43.7%	27.1%	36.3%	
Other Race	33.6%	36.3%	30.1%	

**Table 3:** Consumer views regarding point-of-sale restrictions on tobacco products in pharmacies

	**Allowed**	**Allowed, but only if the products are hidden from view**	**Not allowed at all**	**p**
**Overall**, n = 2,993	43.1%	25.6%	31.3%	
**Smoking status**				p < .001
Current smokers	67.2%	18.1%	14.6%	
Nonsmokers	38.0%	27.3%	34.7%	
**Children**				p = .002
Child in home	41.2%	29.2%	29.6%	
No children in home	44.3%	23.4%	32.4%	
**Age**				p < .001
Age 18-29	43.9%	25.3%	30.8%	
Age 30-44	47.0%	26.0%	27.0%	
Age 45-59	44.9%	27.1%	27.9%	
Age 60+	36.2%	22.9%	40.9%	
**Region**				p < .001
Northeast	36.5%	25.4%	38.0%	
Midwest	49.5%	23.9%	26.6%	
South	43.8%	22.7%	33.5%	
West	41.2%	30.0%	28.7%	
**Rural/Urban**				ns
Rural	44.9%	24.5%	30.6%	
Urban	43.1%	26.1%	30.9%	
**Sex**	*ns*			p < .001
Male	50.2%	24.1%	25.6%	
Female	36.5%	27.0%	36.6%	
**Race**	p < .01			p < .001
White	47.8%	24.6%	27.7%	
African-American	35.9%	24.4%	39.7%	
Other Race	30.7%	29.8%	39.5%	

**Table 4:** Logistic regression assessing consumer views regarding point-of-sale restrictions on tobacco products in pharmacies and grocery stores

	**In grocery stores, tobacco products should be allowed, but only if the products are hidden from view or not allowed at all**	**In pharmacies, tobacco products should be allowed, but only if the products are hidden from view or not allowed at all**
	**N = 2,908**	**N = 2,909**
Current smokers	Ref	Ref
Nonsmokers	3.9 (3.1-4.8)	3.3 (2.7-4.0)
Child in home	1.3 (1.1-1.6)	1.3 (1.1-1.6)
No children in home	Ref	Ref
Age 18-29	Ref	Ref
Age 30-44	.9 (.7-1.1)	.8 (.7-1.0)
Age 45-59	1.1 (.8-1.3)	1.1 (.9-1.4)
Age 60+	1.6 (1.2-2.1)	1.6 (1.3-2.1)
Northeast	1.4 (1.1-1.8)	1.2 (1.0-1.6)
Midwest	.9 (.8-1.2)	.8 (.7-1.1)
South	.9 (.8-1.1)	.9 (.7-1.1)
West	Ref	Ref
Rural	1.9 (.8-1.2)	.9 (.7-1.3)
Urban	Ref	Ref
Male	Ref	Ref
Female	1.6 (1.4-2.0)	1.6 (1.4-1.9)
White	Ref	Ref
African-American	1.3 (1.0-1.7)	1.7 (1.3-2.3)
Other	2.1 (1.7-2.5)	2.2 (1.7-2.7)

Regarding respondents preferences for obtaining medications; 41.7% of respondents would prefer to get their medications from a pharmacy that doesn’t also sell tobacco products^c^. Significantly more non-smokers (48.7%) than smokers (11.5%) and more females (45.7%) than males (37.4%) reported they ‘would prefer to get medications from pharmacies that did not sell tobacco’ (p < .05). Subsequent analyses indicated that this preference was most common among never smokers (52.7%), whereas former smokers (39.7%) were more likely to prefer to get medications from pharmacies that did not sell tobacco than current smokers (11.5%). Among those adults who did not prefer to obtain medications from pharmacies that did not sell tobacco products, approximately one-quarter (23.2%) would change their preference if their doctor recommended this course of action. Nonsmokers (25.8%) were more likely than smokers (15.9%) to report a willingness to follow this recommendation (p < .05).

## Discussion

Most consumers support banning sales of tobacco in grocery stores and pharmacies or allowing sales only if the products are hidden from direct view. Consumers’ support for restricting tobacco product sales in grocery stores and pharmacies was consistent with past local market studies. In a survey of California consumers, Hudmon and colleagues (2006) found that the majority of respondents were not ‘in favor of sales in pharmacies’ [15]. A survey of San Francisco residents following the implementation of legislation prohibiting the sale of tobacco products in pharmacies also found support the ban [30]. Similarly, consumers from a 2011 focus-group study supported the decision of a few California-based independent pharmacies and grocery stores to ban tobacco sales in their businesses [29].

Significantly higher percentages of smokers expressed a more permissive view regarding the sale of tobacco products in pharmacies as well as grocery stores, than non-smokers. This observation is similar to what has been previously noted in other tobacco control policy areas such as ban of tobacco sales in vending machines and prohibition of smoking tobacco on airlines [28,38,39]. The recent evidence documenting an increase in the sale of cigarettes in these venues over the years [12], even as total cigarette sales have fallen, suggests that ubiquitously present community-based pharmacies must compete well on accessibility or price. Future research should explore whether smokers’ views of the primary role of pharmacies, either as a convenience store or a venue for healthcare delivery, vary significantly from those of non-smokers; and if this might partially explain the difference in pharmacy-based tobacco sale preferences between the two groups. It also remains to be seen if smokers might be less concerned than non-smokers about the potential impact that the ubiquitous presence of tobacco-selling retail outlets might have on perpetuating tobacco consumption for themselves or youths [29].

Males, white adults, adults between the age range of 30–59, and those residing in the Midwest or Southern region of United States were least likely to support a ‘ban’ or ‘hidden from view’ approach to tobacco sales in pharmacies and grocery stores. Perhaps differences in prevalence rates of smoking account for these findings [40]. It is also possible that regional differences in statewide funding for tobacco control [41], low state cigarette taxes, as well as weaker tobacco control policies in the in the Southern region [42,43] play a role in consumers’ opinions. Future research should explore probable reasons for this distinction, including whether any higher level societal norm(s), such as resistance to government regulations, might influence the perceived acceptability of tobacco sales among these groups of consumers. It will also be interesting to see if and how these permissive opinions might alter with the changing regulatory environment [44].

About 40% of respondents indicated that they would ‘prefer to get their medications at a pharmacy that did not sell tobacco products’. This appears slightly at odds with what McDaniel and colleagues (2011) learned in focus-groups of California-based consumers [29], who indicated that a pharmacy’s decision to stop selling tobacco products would not influence their decision to shop there. Similarly, in another study, a large majority of consumers reported that even if a pharmacy decided to drop sales of tobacco products they would still shop there just as often; although a small percentage reported they would shop there more often as a result [15]. Thus the research findings are inconclusive. Although some consumers indicated that they would prefer to get their medicines from pharmacies not selling tobacco products, it is possible that consumers in general are indifferent toward the policy. Furthermore, a large majority of those who reported their preference for getting medications at pharmacies not selling tobacco products were non-smokers further highlighting the divide in opinions based on smoking status. Although current smokers were substantially less likely to hold this preference, it is worth noting that the active addiction to smoking itself may be influencing the attitudes rather than a innate predisposition. Given the attitudes of former smokers, we would expect opinions of smokers to shift into the majority view favoring more strict regulation of tobacco product sales as more smokers quit.

Finally, among those respondents who reported *not* preferring to get medications from a pharmacy that doesn’t also sell tobacco products, about one quarter reported that they would adhere to a recommendation from their physician to do so. This pliability suggests that there might be a role for physicians to help shift perceptions about tobacco sales in pharmacies.

The majority of respondents favored increasing the restriction on sales of tobacco products in grocery stores and pharmacies, and many consumers would prefer to obtain their medications form pharmacies that do not sell tobacco products. The pharmacy profession shares this position. Practicing pharmacist, student, and professional organizations have historically held similarly restrictive views of the sale of tobacco products in pharmacies [13-20]. Further, pharmacy advocates have been diligently working on identifying innovative tobacco treatment models that can be feasibly implemented in community pharmacy settings [45,46]. Unfortunately however, not only do many pharmacies (especially chains) continue to sell cigarettes, but in fact the sales have risen by 23% between 2005 and 2009 [12], now accounting for 5% of total cigarette sales. Clearly, this practice is not supported by most pharmacists and consumers, and yet tobacco products continue to remain in pharmacies despite decades of efforts on the part of pharmacist professional associations to remove them.

This is the first study to have employed a nationally representative survey sample to gather consumers’ opinions on the sale of tobacco products in pharmacies and grocery stores. Although the mixed-mode frame applied for this survey substantially reduces concerns of the increasing bias in RDD surveys arising from noncoverage due to wireless substitution, this study is subject to at least four limitations. although the mixed-mode design substantially reduced noncoverage bias compared to an RDD design by including respondents who did not have a landline telephone in their home, it is possible that the dual sampling frame did not entirely eliminate noncoverage issues. Second, ongoing engagement might lead to panel conditioning, and thereby reduce data reliability if respondents develop a “time-in-sample bias” due to increased experience with completing surveys. However, results from the primary analyses did not change with the inclusion of a variable that measured time on the panel. (For the mode 2 frame, analyses presented in each of the tables were replicated with the inclusion of a variable that measured length of time on the panel. The pattern of results did not change, and no evidence of a “time-on-panel bias” was detected.) Third, there was little difference in consumers’ perceptions of tobacco sales in pharmacies and grocery stores. The survey instruments did not include measures to assess perceptions of community pharmacy. Therefore it is not possible to determine whether this similarity is due to a lack of awareness of the concept of community pharmacy among U.S. consumers or strong general support for tobacco control policies that restrict tobacco sales. Finally, the survey items did not define the terms grocery store, pharmacy, or tobacco products. It is possible that respondents held different interpretations for these concepts.

## Conclusions

There is rapidly growing emphasis and interest in regulating the sale of tobacco products in retail outlets, particularly pharmacies. Poor pharmacy-based access to over-the-counter nicotine replacement products [47] as compared to easier access to tobacco in neighborhoods with higher percentage of smokers [48-50] further highlights the need to regulate tobacco retailing by restricting retail licensing [22]. This was the first national level survey to assess consumer opinions on banning the sale of tobacco products in pharmacies and grocery stores. It is very encouraging that consistent with the views of public health advocates, regulators and the medical community, consumers are also largely supportive of more restrictive policies.

## Endnotes

^a^The survey item did not differentiate pharmacies and drug stores. Drug stores were included, in case respondents were unfamiliar with the term pharmacies.

^b^The survey instrument did not provide an explicit ‘no opinion’ or ‘don’t know’ option. However, respondents were instructed to skip any question that they did not want to answer or for which they did not have an opinion. For grocery stores, 2.1% had no opinion; for pharmacies, 2.3% had no opinion.

^c^For this item, 7.4% of respondents had no opinion. Nonsmokers, older adults, adults in the West region, and females were more likely to report no opinion.

## Competing interests

The authors declare that they have no competing interests.

## Authors’ contributions

PP and RM developed the concept for this study. RM and JW developed the survey instrument and methodology. RM obtained IRB approval from Mississippi State University, coordinated the data collection, and performed the statistical analyses. All authors contributed to the drafting of the manuscript, and reviewed and approved the final draft. All authors read and approved the final manuscript.
